# Bacterial and fungal co-infections among ICU COVID-19 hospitalized patients in a Palestinian hospital: a retrospective cross-sectional study

**DOI:** 10.12688/f1000research.74566.1

**Published:** 2022-01-11

**Authors:** Hani A. Naseef, Ula Mohammad, Nimeh Al-Shami, Yousef Sahoury, Abdallah D. Abukhalil, Mutaz Dreidi, Ibrahim Alsahouri, Mohammad Farraj

**Affiliations:** 1 Pharmacy, Birzeit University, Ramallah, Palestine, 14, Palestinian Territory; 2 Department of Nursing, Birzeit University, Ramallah, Palestine, 14, Palestinian Territory; 3 Infectious Disease Department, Beit Jala Govermental Hospital, Ministry of Health, West Bank, Palestinian Territory; 4 Master Program in Clinical Laboratory Science, Birzeit University, Ramallah, Palestine, 14, Palestinian Territory

**Keywords:** COVID 19, Co-infection, Palestine, Iron supplements, ICU

## Abstract

**Background:** Diagnosis of co-infections with multiple pathogens among hospitalized coronavirus disease 2019 (COVID-19) patients can be jointly challenging and essential for appropriate treatment, shortening hospital stays and preventing antimicrobial resistance. This study proposes to investigate the burden of bacterial and fungal co-infections outcomes on COVID-19 patients. It is a single center cross-sectional study of hospitalized COVID-19 patients at Beit-Jala hospital in Palestine.

**Methods: **The study included 321 hospitalized patients admitted to the ICU between June 2020 and March 2021 aged ≥20 years, with a confirmed diagnosis of COVID-19 via reverse transcriptase-polymerase chain reaction assay conducted on a nasopharyngeal swab. The patient's information was gathered using graded data forms from electronic medical reports.

**Results:** The diagnosis of bacterial and fungal infection was proved through the patient’s clinical presentation and positive blood or sputum culture results. All cases had received empirical antimicrobial therapy before the intensive care unit (ICU) admission, and different regimens during the ICU stay. The rate of bacterial co-infection was 51.1%, mainly from gram-negative isolates ( *Enterobacter* species and *K.pneumoniae*). The rate of fungal co-infection caused by *A.fumigatus* was 48.9%, and the mortality rate was 8.1%. However, it is unclear if it had been attributed to SARS-CoV-2 or coincidental.

**Conclusions:** Bacterial and fungal co-infection is common among COVID-19 patients at the ICU in Palestine, but it is not obvious if these cases are attributed to SARS-CoV-2 or coincidental, because little data is available to compare it with the rates of secondary infection in local ICU departments before the pandemic. Comprehensively, those conclusions present data supporting a conservative antibiotic administration for severely unwell COVID-19 infected patients. Our examination regarding the impacts of employing antifungals to manage COVID-19 patients can work as a successful reference for future COVID-19 therapy.

## Introduction

Severe Acute Respiratory Syndrome Coronavirus 2 (SAR-CoV-2) is a highly contagious novel viral pathogen that provokes an immediate spread among hospitalized patients with Community-Acquired Pneumonia (CAP) ^1^; extending from mild symptoms in approximately 84% of patients to life-threatening hypoxic conditions necessitating admittance into the Intensive Care Unit (ICU) and oxygen support. ^2^ Additionally, it causes multi-organ failure that involves sepsis and thromboembolic complications, which progresses into an acute kidney and cardiac injury. ^3^ On March 11 ^th,^ 2020, the World Health Organization (WHO) deemed coronavirus disease 2019 (COVID-19) a pandemic disease, due to its unusual transmission speed and the wide-scale of infection. ^4^

The bacterial and fungal co-infections are frequently recurring due to respiratory viral diseases. For example, most deaths in the Spanish flu pandemic in 1918 were due to subsequent bacterial infection. ^5^ The possibility for co-infections raises concerns as it hinders COVID-19 management, worsens prognosis, and might increase the fatality rate. The reported prevalence of bacterial co-infection varies between studies. Previous studies showed low prevalence of early bacterial coinfection. ^6^^,^^7^ On the other hand, data from France pointed to a high prevalence of early bacterial coinfection during severe COVID-19 pneumonia. ^8^

Throughout the pandemic, evaluation of gathered specimens from hospitalized COVID-19 patients reported the following as the most prevalent organisms that induce co-bacterial infection: *S. aureus, S. epidermidis, H. influenzae, Streptococcus* spp., *E. coli, K. pneumoniae and P. aeruginosa.* ^9^ Therefore, many COVID-19 treatment protocols include empirical antibiotic therapy to cover suspected organisms. Relatively, in a systematic review, 30 studies were summarized including 3834 COVID-19 patients. Overall, only 7% of the hospitalized patients were confirmed to have bacterial co-infection with one or more pathogens, primarily in seriously ill patients. ^10^ On the contrary, diagnosis and/or treatment of fungus infections in COVID-19 patients are neglected or delayed and the actual prevalence of fungal co-infection is yet to be established. Few studies were conducted in this regard; one systematic review meta-analysis research was carried out in China on 2780 confirmed SAR-CoV-2 participants from nine relevant articles. After fungal culturing at admission, 0.12%- 0.15% of the cases were positive for fungal infection and Asian patients were more likely to acquire fungal co-infection compared to the patients in the studies from the U.K. The infecting fungi include *Aspergillus, Candida, Cryptococcus neoformans,* and *Pneumocystis.* ^11^

The exact mechanism of microbial co-infection is inadequately understood. Virologists assumed that the viruses attach and penetrate the host's airway epithelial cells creating an inflammatory response, desensitizing Toll-like receptors and leading to cellular apoptosis in numerous mechanisms. ^12^ In addition, it debilitates the body's defense, induces cellular dysfunction and death. ^12^

Viruses aid in proliferation, colonization, and invasive infection of opportunistic nosocomial pathogens or respiratory tract normal flora, via hindering the innate immune response, compressing airway mucus, and disrupting the cilia. This will then allow for the virus to spread and adhere to more sites. ^3^ Furthermore, various researchers ^13^^,^^14^ highlighted the synergy regulations among viruses and bacteria; both are jointly advantageous, aggravating clinical outcomes. If this cooperation is confirmed, then the management of some viral infections with antibiotics becomes further necessary. ^15^

COVID-19 has been chiefly linked to high levels of inflammatory markers, such as elevated levels of C-reactive protein and procalcitonin. Accordingly, the similarity between Sar-CoV-2 and bacterial infections in radiological infiltrates and laboratory findings makes it challenging in daily medical practice to provide a precise diagnosis. ^16^ However, according to current studies, most COVID-19 patients did not have bacterial or fungal co-infections upon admission or through the hospital stay, even though they received antibiotics upon admission or before diagnosis. ^17^ Furthermore, many factors advocated escalation of antimicrobial consumption in the pandemic: lack of specific antiviral agents, the overload to the health services’ capacities, saturated laboratories, and diagnosis uncertainty. ^18^ As a result, clinicians are obliged to prescribe broad-spectrum multi-antibiotic regimens in order to treat all the possible infected pathogens. Therefore, this study aimed to investigate and evaluate bacterial and fungal co-infection burden and prevalence among COVID-19 patients admitted to a treatment facility at Beit-Jala hospital in Palestine. In addition, it reveals the etiology of the infection, provides the antimicrobial stewardship that was used in the hospital and gives insight into the association of clinical outcomes with several factors and comorbidities.

## Methods

### Ethics and consent

The current research is based on data extraction from electronic medical reports and as such it is not considered human subject research. Neither patients nor the public were involved in the design, conduct, or reporting of this research. The research was done retrospectively and without patient involvement. The study was approved by the Scientific Research Ethics Committee of Birzeit University on 27-2-2021 and by Beit-Jala hospital on 10-3-2021. The permission to use the generated data was obtained from Beit Jala Governmental Hospital laboratory administration.

### Study design and study population

This single-center cross-sectional study was conducted on patients aged 20 years and older in Beit-Jala hospital (as patients under 20 are not admitted to this hospital), one of the central institutes for treating SAR-CoV-2 patients in Palestine. A total of 458 records were screened. The records were for critically ill patients, admitted to the ICU between June 2020 and March 2021, with a confirmed diagnosis of COVID-19 via reverse transcriptase-polymerase chain reaction assay (RT-PCR) conducted on nasopharyngeal swab specimens. 321 patients with positive cultures for bacteria or fungi were included in the study, and patients with negative cultures were excluded.

### Data extraction and analysis

Data extraction was done manually by one researcher between the 15 ^th^-29 ^th^ of March 2021, and verified by a second researcher, using graded data forms from electronic medical reports. The obtained data were socio-demographics, chronic comorbidities, laboratory findings (CRP, leukocytes, blood oxygen saturation), duration of the ICU stay and if they had taken an iron supplement. The bacterial or fungal co-infection was proved through clinical presentation and positive blood or sputum testing via Laboratory Information System. Before and during the ICU admission, antimicrobial utilization was recorded, and the reports were double-checked for accuracy and completeness.

Data was analyzed and socio-demographic and clinical characteristics were summarized using relevant descriptive statistics, and categorical variables as percentages and frequencies. Pearson’s Chi-square test was performed to determine the association between the main parameters, which were the ICU residency duration and the other factors such as age, gender, iron supplements, antibiotics administration before and during the ICU admission, smoking habits, and comorbidities. Data was analyzed and presented using SPSS version 22.0.

### Definitions

Co-infection is a sequela that occurs during or after the primary causative pathogen or its treatment. The isolated bacteria or fungi obtained from COVID-19 patients should be clinically relevant and not microbiota or contaminants. All the microbiological samplings were conducted upon the hospital`s entry and before the ICU admittance, and only patients holding positive bacterial or fungal outcomes were admitted into the unit. In combination with decision-making, a scope of tests should be met to provide permission on who necessitates access, including computerized tomography (CT) scans to reveal ground-glass opacification, which correlated with pneumonia. In addition, supplementary laboratory outcomes were crucial in making the diagnosis, such as C-reactive protein (CRP) level, Oxygen saturation (SpO2), Leukocyte numbers and body temperature. Patients were immediately admitted to the ICU if two or more of the above matched positive isolates test and their CT-scan showed pneumonia.

To determine the status of each patient, laboratory findings must be evaluated. For example, CRP values that ranged between 4.5-8.5 mg/dL were rated as moderate and ≥8.5 mg/dL was rated as severe. Leukocytes results in the 4.6-8 g/L range were considered mild, while >8 g/L indicated seriously ill patients. Normal oxygen saturation was considered ≥95%, but in COVID-19 patients, an oximeter reading at rest of 90% or lower was classed as a severe case. The body temperature was deemed high if it was between 38-39 °C and very high if it was >39 °C.

## Results

### Presenting demographics

A total of 458 moderate-severe ill patients were screened and evaluated, and of those 321 participants were included in this study. The patients’ demographics and clinical characteristics are presented in Table 1. Overall, 48.9% (170) were 41-64 years of age and 50.5% (162) were males. Prevailing chronic medical conditions included diabetes mellitus at 77.9% (250), atrial hypertension at 66.4% (213), obesity at 36.8% (118), cancer at 20.6% (66), and coronary heart disease at 23.1% (74). 3.7% of patients did not have past medical history of any comorbidity. 58.6% were not smokers, and 62.6% were supplemented with Iron 376 mg/day intravenously for three days. Additionally, all the participants in this study had received N-acetyl-cystine at 1200 mg/day, which began when they were admitted and continued until they left.

**Table 1.** Demographics, medical history and antibiotic regimens. (N 321; ICU: intensive care unit; SpO _2_: oxygen saturation).

**Gender**	Male		162 (50.5%)
	Female		159 (49.5%)
**Age (years)**	20-40		54 (16.8%)
	41-64		157 (48.9%)
	>65		110 (34.3%)
**Smoking habits**	Yes		133 (41.4%)
	No		188 (58.6%)
**Presence of comorbidities**	Diabetes mellitus		250 (77.9%)
	Atrial hypertension		213 (66.4%)
	Obesity		118 (36.8%)
	Coronary heart disease		74 (23.1%)
	Cancer		66 (20.6%)
	No personal history		12 (3.7%)
**Laboratory findings on day of ICU admission**	CRP	Moderate	175 (54.5%)
		High	146 (45.5%)
	Leukocytes	Moderate	79 (24.6%)
		High	242 (75.4%)
	SpO _2_	>90%	71 (22.1%)
		<90%	250 (77.9%)
	Fever	High	94 (29.3%)
		Very high	227 (70.7%)
**Microbiological findings**	*A. fumigatus*		157 (48.9%)
	*Enterobacter*		80 (24.9%)
	*K. pneumoniae*		54 (16.8%)
	*P. aeruginosa*		10 (3.1%)
	*vancomycin-resistant enterococci*		10 (3.1%)
	*S. aureus*		9 (2.8%)
	Invalid data		1 (0.3%)
**Antibiotic regimen before admittance into the ICU**	Ceftriaxone (2 g/day)		223 (69.5%)
	Ampicillin/sulbactam (12 g/day)		98 (30.5%)
**Antibiotic regimen during the ICU stay**	Meropenem+ vancomycin (2 g + 1 g/day)		138 (43.0%)
	Piperacillin/tazobactam + Levofloxacin (13.5 g+ 500 mg/day)		114 (35.5%)
	Fluconazole (200 mg/day)		69 (21.5%)
**Iron supplement [376 mg] administration**	Yes		201 (62.6%)
	No		120 (37.4%)
**Duration of ICU stay (days)**	1-6		234 (72.9%)
	7-11		61 (19.0%)
	Death		26 (8.1%)

### Bacterial and fungal co-infection

The overall mortality rate in this study was 8.1%, ^26^ while 72.9% (234) of the patients remained in the ICU for 1-6 days. Each patient was infected with one type of bacteria or fungi, which presented as a respiratory infection, bacteremia, and skin infections. A total of six different pathogens were detected in the samplings and considered as the infecting organisms. The most abundant types were *Aspergillus fumigatus* 48.9% and *Enterobacter* 24.9%, as shown in Table 1.

### Patterns of antibiotic use

In terms of antibiotic prescription patterns, through the first 24 hours of hospital admission and based on per case specificity, the proper liver or kidney function tests had been conducted in all cases, in order to determine the appropriate regimen for treating bacterial or fungal infections. Before the ICU access, the treatment span was six days on average; 69.5% (223) received ceftriaxone 2 g/day, and 30.5% (98) patients had received ampicillin and sulbactam 12 g/day; both regimens were administered intravenously (IV). Upon ICU admission, every patient had been given one kind of IV antibiotic regimen throughout the stay; 43.0% (138) took meropenem 2 g/day and vancomycin 1 g/day and 35.5% (114) were given piperacillin/tazobactam 13.5 g/day and levofloxacin 500 mg/day. By January and after getting financial support from the Ministry of Health, patients infected with *A. fumigatus* started therapy with fluconazole 200 mg/day. Since then, 100% of the patients with fungal infection were successfully treated. Considering different regimens had notable distinct outcomes, analysis was performed to determine the association linking antimicrobial therapy class and the ICU stay or death ( Table 2). After hospital discharge, patients taking antibacterial medications were switched to oral azithromycin. Patients treated with Fluconazole IV for two days switched to oral Fluconazole 150 mg/week.

**Table 2.** Impact of different non-significant values on the duration in the ICU. (N 321; ICU: intensive care unit; SpO _2_: oxygen saturation).

Parameter		Duration 1-6 days	Duration 7-11 days	Death	*P* value
Age (years)	**20-40**	41 (75.9%) *	10 (18.5%)	3 (5.6%)	0.413
	**41-64**	119 (75.8%)	28 (17.8%)	10 (6.4%)	
	**>65**	74 (67.3%)	23 (20.9%)	13 (11.8%)	
Diabetes	**Yes**	180 (72.0%)	49 (19.6%)	21 (8.4%)	0.794
	**No**	54 (76.1%)	12 (16.9%)	5 (7.0%)	
Hypertension	**Yes**	156 (73.2%)	43 (20.2%)	14 (6.6%)	0.317
	**No**	78 (72.2%)	18 (16.7%)	12 (11.1%)	
Obesity	**Yes**	81 (68.6%)	25 (21.2%)	12 (10.2%)	0.383
	**No**	153 (75.4%)	36 (17.7%)	14 (6.9%)	
Smoking	**Yes**	103 (77.4%)	25 (18.8%)	5 (3.8%)	0.052
	**No**	131 (69.7%)	36 (19.1%)	21 (11.2%)	
CRP	**Moderate**	128 (73.1%)	33 (18.9%)	14 (8.0%)	0.994
	**High**	106 (72.6%)	28 (19.2%)	12 (8.2%)	
Leukocytosis	**Moderate**	59 (74.7%)	18 (22.8%)	2 (2.5%)	0.089
	**High**	175 (72.3%)	43 (17.8%)	24 (9.9%)	
SpO _2_	**<90**	188 (75.2%)	40 (16.0%)	22 (8.8%)	0.032
	**>90**	46 (64.8%)	21 (29.6%)	4 (5.6%)	
Fever	**High**	71 (75.5%)	19 20.2%)	4 (4.3%)	0.265
	**Very high**	163 (71.8%)	42 (18.5%)	22 (9.7%)	

* The percentages indicate proportion of patients within each category in any duration.

Table 2 reports no significant association between the ICU stay duration with age, laboratory test results or having a chronic disease. A significant association was found between the residency duration and SpO _2_ ( *P*=0.032): patients whose SpO _2_ levels were <90% (188; 75.2%) were admitted in the ICU for a shorter duration (1-6 days) compared to patients whose SpO _2_ read >90% (46; 64.8%).

Table 3 shows a significant association between the ICU residency duration and the infecting isolates ( *P*=0.008). The patients suffering from fungal co-infection (126; 80.3%) spent fewer days in the ICU than patients with bacterial co-infection (108; 65.9%).

**Table 3.** Impact of different significant values on the duration in the ICU (intensive care unit).

Parameter		Duration 1-6 days	Duration 7-11 days	Death	*P*
**Ampicillin/sulbactam**		52 (53.1%) **	29 (29.6%)	17 (17.3%)	<0.001
**Ceftriaxone**		182 (81.6%)	32 (14.3%)	9 (4.0%)	
**Piperacillin/tazobactam+levofloxacin**		57 (50.0%)	37 (32.5%)	20 (17.5%)	<0.001
**Meropenem+vancomycin**		108 (78.3%)	24 (39.3%)	6 (4.3%)	
**Fluconazole**		69 (100%)	0 (0%)	0 (0%)	
**Iron Supplement**	**Yes**	199 (99%)	0 (0%)	2 (1.0%)	<0.001
	**No**	35 (29.2%)	61 (50.8%)	24 (20.0%)	
**Fungi**		126 (80.3%)	24 (15.3%)	7 (4.5%)	0.008
**Bacteria**		108 (65.9%)	37 (22.6%)	19 (11.6%)	

** The percentages indicate proportion of patients within each category in any duration.

Regarding the hospital`s therapy strategy, there was a significant association between ICU residency duration and the empirical antibiotics ( *P*<0.001); patients administered ceftriaxone (182; 81.6% of the patients given ceftriaxone) were more likely to stay in the ICU for fewer days compared with patients administered ampicillin/sulbactam (52; 53.1%). In addition, patients who took fluconazole (69; 100%) were significantly more likely to stay for fewer days in the ICU compared to those administered piperacillin and tazobactam with levofloxacin (57; 50%) or meropenem with vancomycin (108; 78.3%) respectively ( *P*<0.001).

Moreover, patients that were administered iron supplements as part of their therapy regimen (199; 99%) were significantly more likely to stay for a shorter duration of time compared with those who were not administered iron (35; 29.2%, *P*<0.001).

## Discussion

In this research, the characteristics of co-infection in 321 severely ill COVID-19 patients were evaluated. This research found a mortality rate of 8.1% in the ICU, compared to a 25.7% mortality rate that was obtained from a systematic review of the emerging literature including 15 studies carried out in countries most affected by the pandemic. ^20^ Despite the absence of antimicrobial stewardship in urgent response, the hospital's initiation of drastic empirical administration was crucial for preventing or treating suspected infections. In addition, the hospital’s guideline adherence for empiric antimicrobial therapy and the other drugs was adequate.

COVID-19 patients are at risk of fungal infections during the latter stages of the disease, due to seriously damaged alveoli and the decline in leukocyte counts. ^21^ Based on the knowledge of SARS in 2003 and the invasive Aspergillosis, it is vital to consider the possibility of a life-threatening fungal infection accompanying COVID-19. At the beginning of blood/sputum culturing, the number of cases infected by *A. fumigatus* was 157. Initially, those patients were given antibacterial regimens before and during their ICU stay; because fluconazole was costly to obtain. By January, confirmed fungal infections were treated with fluconazole. This had reduced the average ICU stay from six days to less than two days, and the mortality rate from *A. fumigatus* decreased from 4.5% to 0%. Throughout the pandemic, specialists realized the risks of fungal co-infection. Therefore, the French High Council for Public Health recommended that clinicians should concentrate on fungal infections in COVID patients, particularly in severe cases of the disease. ^21^

Bacterial co-infection was recorded in 51.1% of cases, with higher mortality rates among *Enterobacter* infected patients; this may be due to its known drug resistance. It should be noted that normal oral flora colonization or contamination may occur, particularly in the sputum specimens. In general, the empirical treatment with ceftriaxone or ampicillin/sulbactam showed minimal benefits to the clinical status. However, we observed a better prognosis with ceftriaxone than ampicillin/sulbactam, with mortality rates at 4% and 17.3% respectively; this association needs more investigation.

The wide-spectrum antimicrobial regimens are administered once the bacterial co-infection is confirmed and the patient is admitted into the ICU. Unfortunately, there is no available data to compare COVID-19 patients who received antibacterial agents with those who did not, which could help to determine the therapeutic efficacy. However, a regimen of piperacillin with tazobactam and levofloxacin resembles meropenem and vancomycin in terms of clinical outcomes and management rate. This counters a previous study that revealed a higher rate of mortality and organ failure in patients administered meropenem. ^17^ Furthermore, after the recovery and discharge from the hospital, all outpatients were prescribed azithromycin, which also has antiviral activity and may decrease the incidence of acute organ failure. ^17^

Earlier examinations illustrated that multiple antibiotic administrations did not alter the disease results or had joined with higher mortality rates. ^22^ In our study, it appeared to induce a good prognosis. Ceftriaxone, meropenem, and vancomycin regimens have been linked with more favorable outcomes and lower mortality rates. Until solid evidence is available, antimicrobials should be maintained for critical cases and constantly re-evaluated based on the patient's progress. Most importantly, the therapy must only cover the suspected or confirmed bacterial infection.

Antibiotic resistance and allergic reactions are multifactorial issues, and the pandemic has impacted the health system and provided a good environment for bacteria to become resistant to antimicrobials. In our study, all COVID-19 patients were admitted to the ICU and received at least four diverse types of broad-spectrum antibacterial agents; in addition, the accessibility of over-the-counter antimicrobials had undeniably grown through the pandemic. ^19^ This inappropriate behavior will lead to the evolution of high levels of antibiotic-resistant bacteria.

The unusual incidence of bacterial or fungal infections in our study differs from other published studies. ^11^^,^^23^ This significant finding is due to the participant characteristics of critically ill patients receiving therapy via invasive catheters and being at risk of infection by nosocomial pathogens. Moreover, the unusual rate of infection has been credited to the exhaustion in the clinical care system creating a prolonged hospital exposure, principally in the major waves of the virus.

In our study, 100% of the patients were severe-critical ill. Therefore, it is necessary to differentiate between them and mild-moderate patients and prioritize the ICU admittance. Fever was the notable symptom in SARS-CoV-2 infection identified at the early stages of the disease. Regarding laboratory findings, leukocytosis occurred in critically ill patients with distinct grades, and it was observed in 11.4% severe COVID-19 cases. ^24^ Leukocytosis aids in the disease progress evaluation, and its intensity reflects the severity of COVID-19 infection; it is exacerbated by the cytokine storm and inflammatory mediators that drive apoptosis and pulmonary microvascular destruction, creating alveolar oedema. The increased level of CRP is a diagnostic biomarker for coronavirus in distinguishing moderate and critical patients. The pathogenesis of COVID-19 involves a vigorous inflammatory response that manages a dysregulated flood of immune cells and signaling molecules. ^25^ In general, viral respiratory infections have been linked to hypoxia. COVID-19 patients are considered hypoxic if their oxygen saturation level is <90%, while the normal SpO2 is ≥95% at rest in healthy people. Hypoxia is a fearful condition that is monitored and adjusted by mechanical ventilation. ^26^ Although several studies ^27^^,^^28^ reported links between laboratory findings and clinical outcomes, our cases did not show a significant link.

Throughout the coronavirus pandemic, the issues of tobacco smoking and the risk of infection were constantly reviewed. Published research ^29^ indicated that smokers are twice as likely to develop severe COVID-19 as nonsmokers, as smoking tobacco induces alterations in the respiratory tract and cell-mediated immune response. ^29^ Furthermore, current smokers have higher ACE-2 gene expression than non-smokers; this gene is responsible for ACE-2 receptors production, which COVID-19 attaches to and penetrates the cells, thus raising the chance of infection. ^30^ Our study did not find an association between smoking habits and the duration of ICU stay or clinical prognosis.

Numerous investigations ^31^^,^^32^ found that older COVID-19 patients aged ≥50 years were correlated with a higher risk for severe signs, atypical presentation, the opportunity for co-infections, and higher fatality rates than patients <50 years of age. The rate usually increases rapidly with age, which is not surprising due to the drop in natural immunity with aging and associated comorbidities. In addition, it is believed that older people are prone to adverse drug reactions. ^32^ In general, people of older ages are more prone to becoming infected with the COVID virus than younger people. ^32^ A review reported that the fatality rate is doubled in males compared to females; it stated that male patients may have higher ACE-2 enzyme activity, directed by male sex hormones, contributing to more risk factors for SARS-CoV-2 infection and worsening clinical outcomes. ^33^ Additionally, the smoking rate is higher in males. ^33^

Effects of comorbidities such as hypertension, diabetes mellitus, coronary heart disease, and cancer were compared between survivors and non-survivors of COVID-19. Participants with comorbid conditions have a significantly greater fatality rate compared to participants without comorbid conditions, because they have decreased natural immunity and are poly-pharmacy patients. ^34^^,^^35^ There was no association between age, gender, or comorbidities with the clinical outcomes and ICU stay duration in our study.

Previous reports had described mild anemia in COVID-19 patients at the ICU; due to severe inflammation which consumes iron, ^36^ the innate immune system limits the bioavailability of iron in order to diminish viral replication and infection. Accordingly, iron absorption from the diet is reduced, and the liver generates hepcidin which blocks the carrying of iron out of the cell. Accordingly, iron supplementation may exacerbate the disease and increase the inflammatory process. ^37^ However, no solid investigations were performed on the relation between iron and clinical results in COVID-19 patients. In our study, 62.6% of the patients had received IV iron supplement for three days, based on a randomized clinical trial, and 99% of them had improved and stayed for a shorter time (1-6 days) in the ICU than those who did not receive IV iron; thus it can be suggested that the iron provided an enhancement to immunity. Therefore, there is a strong association between low serum levels of iron and an increased infection rate and morbidity.

COVID-19 produces oxidative stress irregularity, and this process has been enhanced by glutathione reduction. Thiones are synthesized in a multi-step process involving N-acetyl-cysteine (NAC). Thiones are also ACE-2 blockers, thereby diminishing SARS-CoV-2 penetration into the cell. In a placebo-controlled study, NAC administration had reduced plasma inflammatory biomarker levels via several mechanisms. Besides antioxidant activity, NAC has vasodilator activity, especially in the intravenous route. In addition, it has mucolytic properties, inhibits RNA virus replication, and has confirmed protective effects against comorbid conditions, including cardiovascular diseases. ^38^ In our study, patients received NAC 1200 mg/day during the hospital stay, and even after discharge they showed a good prognosis and improvement in their health status.

### Strengths and limitations of this study

This is the first research addressing co-infection among COVID-19 patients in Palestine and offering clinical base recommendations for patient management. Our study shows a high prevalence of fungus and bacterial co-infection among COVID-19 patients admitted to the ICU, which requires appropriate management and attention. The study findings support the importance of assessing fungus co-infection in COVID-19 patients, and the early initiation of an antifungal agent will decrease morbidity and mortality. Our examination regarding the impacts of employing antifungals to manage COVID-19 patients can work as a successful reference for future COVID-19 therapy.

This research has some limitations: first, it is a retrospective cross-sectional study holding obstacles restricting the potential bias and difficulty proofing the relation between the exposure and the outcome. Additionally, it is a single-center study, and its results could vary in another setting. These conclusions present data supporting a conservative antibiotic administration for severely unwell COVID-19 infected patients.

## Conclusion

The COVID-19 pandemic exerts pressure on healthcare professionals, including a high probability of infection. It is eminent to save lives during this pressure, even if it means multi-drug antimicrobial regimens for prevention or treatment. However, the insufficiency of data about antibiotic types, dosage, and duration of treatment through the pandemic continues to be challenging to health care providers. Bacterial and fungal co-infection is common among COVID-19 patients at the ICU in Palestine; it is not evident if these cases are attributed to SARS-CoV-2 or coincidental as there is little available data. Finally, antimicrobial stewardship, appropriate patient assessment, and selecting the appropriate antimicrobial agents for the right patient at the right time will decrease hospital stay, mortality, and health care costs.

## Data availability

### Underlying data

DRYAD: Raw data and SPSS analysis for the article Bacterial and fungal co-infections among ICU COVID-19 hospitalized patients in a Palestinian hospital: Incidence and antimicrobial stewardship. https://doi.org/10.5061/dryad.08kprr53r.

This project contains the following underlying data:

- raw_data.xlsx (raw underlying data)
- Readme.docx (data key)
- variabels_and_Output_final.pdf (SPSS output file)

Data are available under the terms of the Creative Commons Zero “No rights reserved” data waiver (CC0 1.0 Public domain dedication).

## Author contributions

HN Project Administration, Supervision, Writing – Review & Editing, UM and YS Data Curation, Writing – Original Draft Preparation, Formal Analysis, NA Formal Analysis, Writing – Original Draft Preparation, AA Methodology, Writing – Review & Editing, MD Methodology, Writing – Review & Editing, IA Data Curation, MF Writing – Review & Editing. All authors read and approved the final manuscript.
