# Supplementary material for: Bacterial and fungal co-infections among ICU COVID-19 hospitalized patients in a Palestinian hospital: a retrospective cross-sectional study
Source: F1000Res. 2022 May 30;11:30. Originally published 2022 Jan 11. [Version 2] doi: 10.12688/f1000research.74566.2 (PMC9206111; doi:10.12688/f1000research.74566.2)
Supplement: Peer review history

# Peer review: Reviewer response for version 2

**DOI:** 10.5256/f1000research.134315.r139417

**Published:** 2022-06-17

**Authors:** Hamza Maswade

**Affiliations:** 1 College of Pharmacy, Qassim University, Buraydah, Saudi Arabia

All changes are made to the manuscript, no additional comments. I believe that the revised version is acceptable.

Is the work clearly and accurately presented and does it cite the current literature?

Yes

If applicable, is the statistical analysis and its interpretation appropriate?

Yes

Are all the source data underlying the results available to ensure full reproducibility?

Yes

Is the study design appropriate and is the work technically sound?

Yes

Are the conclusions drawn adequately supported by the results?

Yes

Are sufficient details of methods and analysis provided to allow replication by others?

Yes

Reviewer Expertise:

NA

I confirm that I have read this submission and believe that I have an appropriate level of expertise to confirm that it is of an acceptable scientific standard.

# Peer review: Reviewer response for version 2

**DOI:** 10.5256/f1000research.134315.r139416

**Published:** 2022-06-01

**Authors:** Maher Khdour

**Affiliations:** 1 Faculty of Pharmacy, Al-Quds University, Abu Dis, Palestinian Territory

**Bacterial and fungal co-infections among ICU COVID-19 hospitalized patients in a Palestinian hospital: a retrospective cross-sectional study - version 2**

 The authors ****have substantially addressed all questions raised during the review process for the manuscript.

 I can assure readers that the study adds value to the health policy maker and international literature.

Is the work clearly and accurately presented and does it cite the current literature?

Yes

If applicable, is the statistical analysis and its interpretation appropriate?

Yes

Are all the source data underlying the results available to ensure full reproducibility?

Yes

Is the study design appropriate and is the work technically sound?

Yes

Are the conclusions drawn adequately supported by the results?

Yes

Are sufficient details of methods and analysis provided to allow replication by others?

Yes

Reviewer Expertise:

clinical pharmacy, pharmacy practice, pharmaceutical care, medications, drug utilization, disease management.

I confirm that I have read this submission and believe that I have an appropriate level of expertise to confirm that it is of an acceptable scientific standard.

# Peer review: Reviewer response for version 1

**DOI:** 10.5256/f1000research.78339.r130200

**Published:** 2022-05-16

**Authors:** Hamza Maswade

**Affiliations:** 1 College of Pharmacy, Qassim University, Buraydah, Saudi Arabia

The manuscript entitled "Bacterial and fungal co-infections among ICU COVID-19 hospitalized patients in a Palestinian hospital: a retrospective cross-sectional study" is a study to investigate the burden of bacterial and fungal co-infections outcomes on COVID-19 patients. It is a single center cross-sectional study of hospitalized COVID-19 patients at Beit-Jala hospital in Palestine. The study is important and well written; however there are some comments, such as:

- Please, add the duration of ICU stay (day) chronic cross tabulation.
- Please, if it's possible, classify patients according to the number of chronic diseases for each patient.
- Table 1 the author(s) provide the percent for:
  Diabetes mellitus 250 (77.9%)
  Atrial hypertension 213 (66.4%)
  Obesity 118 (36.8%)
  Coronary heart disease 74 (23.1%)
  Cancer

However; in table 2 "Impact of different non-significant values on the duration in the ICU", they did not mention the data for coronary heart disease and for cancer. Please add the data in table 2 and make a discussion for the results concerning patients with coronary heart disease and cancer.

Is the work clearly and accurately presented and does it cite the current literature?

Yes

If applicable, is the statistical analysis and its interpretation appropriate?

Yes

Are all the source data underlying the results available to ensure full reproducibility?

Yes

Is the study design appropriate and is the work technically sound?

Yes

Are the conclusions drawn adequately supported by the results?

Yes

Are sufficient details of methods and analysis provided to allow replication by others?

Yes

Reviewer Expertise:

NA

I confirm that I have read this submission and believe that I have an appropriate level of expertise to confirm that it is of an acceptable scientific standard, however I have significant reservations, as outlined above.

# Response

**Published:** 2022-05-21

**Authors:** Hani Naseef

**Affiliations:** birzeit university, Palestinian Territory

Dear Dr. Hamza, Thank you for your valuable comments. Following are our responses point-by-point to your comments:

 1- Comment 1: Done.

 2- Comment 2: Done, added to table 2.

 3- Comment 3: Done "A significant association was found between the residency duration and having a personal history with no chronic diseases ( *P*= 0.035): (3; 23.1%) of patients with no chronic diseases have died after being admitted to ICU compared to (23; 7.5%) of patients diagnosed with at least one chronic disease."

# Peer review: Reviewer response for version 1

**DOI:** 10.5256/f1000research.78339.r119466

**Published:** 2022-02-01

**Authors:** Maher Khdour

**Affiliations:** 1 Faculty of Pharmacy, Al-Quds University, Abu Dis, Palestinian Territory

**Bacterial and fungal co-infections among ICU COVID-19 hospitalized patients in a Palestinian hospital: a retrospective cross-sectional study**

 The paper and subject are both valuable and are important to the scientific community, especially in this situation during the pandemic Covid-19. The paper is well-written, however some issues were raised during the review process and should be addressed:

- In the abstract it is not clear whether the patients get infected at admission or during their stay in the ICU.
- All results data e.g. "The rate of bacterial co-infection was 51.1%..." should include infected patients / total patients and for mortality rate please state the CI.
- Abstract conclusion (see if it fit the journal regulations as I see it long?).
- Study design should be clearer to readers.
- 458 records screened and 321 positive (this will result in 70% infected); if this is the case, please add the over all co-infection in the sample of 70% to your results.
- Between 4.5-8.5 mg/dL was rated as moderate and ≥8.5 mg/dL was rated as severe. Leukocyte results in the 4.6-8 g/L range were considered mild, while >8 g/L indicated seriously ill patients: this is not mentioned in your results or in the abstract and you can find a good correlation between the severity and co-infection, I think this will make the abstract much better.
- How did you calculate the mortality rate (should be stated in the method section, all causes)?
- Some significant data in tables 2 and 3 worth mentioning in the results of abstract with P values.
- Under the tables, please state the test you used (Chi square) and be sure the abbreviations match too.
- In the discussion (mortality rate 8.1 vs 25% in other developed countries) do you think there is under-reporting or documentation of co-infection in Palestinian hospitals, or is this due to the broad antibiotic used which is restricted in other countries due to resistance concerns?

Is the work clearly and accurately presented and does it cite the current literature?

Yes

If applicable, is the statistical analysis and its interpretation appropriate?

Yes

Are all the source data underlying the results available to ensure full reproducibility?

Yes

Is the study design appropriate and is the work technically sound?

Yes

Are the conclusions drawn adequately supported by the results?

Yes

Are sufficient details of methods and analysis provided to allow replication by others?

Yes

Reviewer Expertise:

clinical pharmacy, pharmacy practice, pharmaceutical care, medications, drug utilization, disease management.

I confirm that I have read this submission and believe that I have an appropriate level of expertise to confirm that it is of an acceptable scientific standard, however I have significant reservations, as outlined above.

# Response

**Published:** 2022-02-03

**Authors:** Hani Naseef

**Affiliations:** birzeit university, Palestinian Territory

Dear Dr. Maher

 Thank you for your valuable comments, when the article has received a second peer review report I will submit a revised version 2 of the article incorporating the changes as you requested.

# Response

**Published:** 2022-05-21

**Authors:** Hani Naseef

**Affiliations:** birzeit university, Palestinian Territory

Dear Dr. Maher Thank you for your valuable comments. Following are our responses point-by-point to your comments:

 1- comment 1: Clarified "with a confirmed diagnosis of COVID-19 via reverse transcriptase-polymerase chain reaction assay conducted on a nasopharyngeal swab and has a positive culture for bacteria or fungi as upon hospital admission".

 2- comment 2: Done in all the text.

 3- comment 3: As per the Article Guidelines: the abstract should be up to 300 words long,

 The abstract of the manuscript fits the journal's instructions.

 4- comment 4: Changed and clarified the text.

 5- comment 5: Clarified  " A total of 458 records of moderate-severe COVID-19 ill patients were screened and evaluated. Of those, 321 patients with positive cultures for bacteria or fungi were included in the study, and patients with negative cultures were excluded".

 6- comment 6: This is already done and written in Table 2, but due to the absence of the association, as the chi-square result revealed, we did not mention it either in the text or in the abstract.

 7- comment 7: Rewritten: "The overall mortality rate for our sample in this study was (26/321*100%= 8.1%)".

 8- comment 8: Done " A significant association was found between the residency duration and SpO 2 ( P=0.032). The results revealed a significant association between ICU residency duration and the empirical antibiotics ( P<0.001)."

 9- comment 9: Done for tables 2 and 3.

 10- comment 10: Done "The mortality rate of 8.1% (26) for patients admitted to the ICU due to Covid-19 coinfections found by this study is much lower than other reported rates of 25.7 % in a systematic review that included 15 studies in different countries. Despite the absence of antimicrobial stewardship in urgent response, the hospital's initiation of drastic empirical administration was crucial for preventing or treating suspected infections. In addition, the hospital’s guideline adherence to empiric antimicrobial therapy and the other drugs was adequate. Furthermore, many factors could affect this finding, including the age and living situations; in our sample, two-thirds of participants were less than 65 years old. In addition, all participants did not live in nursing homes or elderly homes where a higher rate of antimicrobial resistance is expected."
